# Dr. A. Y. P. Garnett

**Published:** 1888-08

**Authors:** 


Dr. A. Y. P. Garnett, of Washington, died at Rehoboth Beach, Del., July 11, 1888, of heart failure. Dr. Garnett gained some notoriety in the spring of 1861 by his secret communications with the Confederate authorities through his father-in-law, ex-Governor Wise, of Virginia, keeping it advised of the movement of troups, etc., in those early attempts of the Washington government to checkmate the rebellion. He was elected president of the American Medical Association in 1887, and presided at the Cincinnati meeting in May last.

